# Annotated Genome Sequences of 16 Lineage 4 *Mycobacterium tuberculosis* Strains from Guatemala

**DOI:** 10.1128/genomeA.00024-18

**Published:** 2018-02-15

**Authors:** Joseph W. Saelens, Dalia Lau-Bonilla, Anneliese Moller, Ana M. Xet-Mull, Narda Medina, Brenda Guzmán, Maylena Calderón, Raúl Herrera, Jason E. Stout, Eduardo Arathoon, Blanca Samayoa, David M. Tobin

**Affiliations:** a Department of Molecular Genetics and Microbiology and Center for Host-Microbial Interactions, Duke University Medical Center, Durham, North Carolina, USA; b Asociación de Salud Integral, Guatemala City, Guatemala; c Division of Infectious Diseases and International Health, Department of Medicine, Duke University Medical Center, Durham, North Carolina, USA; d Hospital General San Juan de Dios, Guatemala City, Guatemala; e Clínica Familiar Luis Ángel García, Guatemala City, Guatemala; f Facultad de Ciencias Químicas y Farmacia, Universidad de San Carlos de Guatemala, Guatemala City, Guatemala

## Abstract

Whole-genome sequencing has resulted in new insights into the phylogeography of *Mycobacterium tuberculosis*. However, only limited genomic data are available from *M. tuberculosis* strains in Guatemala. Here we report 16 complete genomes of clinical strains belonging to the Euro-American lineage 4, the most common lineage found in Guatemala and Central America.

## GENOME ANNOUNCEMENT

Genome sequencing has revealed much about the phylogeography of *Mycobacterium tuberculosis*, wherein discrete genetic lineages of this human pathogen associate with specific regions of the world. While many isolates have been sequenced from a variety of locales, limited genomic sequence data are available regarding *M. tuberculosis* strains in Guatemala. Previously, we reported on the presence of East Asian lineage 2 strains in Guatemala in an urban setting (1). However, throughout Central and South America, Euro-American lineage 4 strains are most common (2). Here we report 16 complete genomes of Euro-American lineage 4 strains. Isolates were collected at the Clínica Familiar Luis Angel García (CFLAG), an HIV-specialized clinic associated with the Hospital General San Juan de Dios in Guatemala City.

Cultures were grown on Lowenstein-Jensen medium. Genomic DNA was extracted using a modified protocol of the ArchivePure DNA cell/tissue purification kit (5 Prime GmbH, Germany). Spoligotyping was carried out using the spoligotyping kit and protocol from Isogen Biosolutions (Ocimum Biosolutions Ltd., India), identifying the isolates as lineage 4 strains. Paired-end 50-bp reads were sequenced on the Illumina HiSeq 2500 platform to depths ranging from 327- to 627-fold coverage. Reads were aligned against the H37Rv reference genome (GenBank accession number NC_000962) using Burrows-Wheeler alignment (3). Variants were called with SAMtools (4) and filtered with VarScan (5) for a minimum read depth of 10, a consensus quality score of 20, and a minimum variant frequency of 0.75. Single nucleotide polymorphisms (SNPs) adjacent to indels and within repetitive regions of the genome were discarded. Neighbor-joining and maximum-likelihood methods of phylogeny construction based on genome-wide SNPs placed the Guatemalan isolates among known lineage 4 strains, confirming the spoligotyping results. Consensus sequences for each isolate were generated using BCFtools (6), and gene annotations were added by the NCBI Prokaryotic Genome Annotation Pipeline.

### Accession number(s).

The genome sequences of the *M. tuberculosis* isolates reported here have been deposited in GenBank under the accession numbers listed in Table 1.

**TABLE 1:** Genome sequence data for 16 lineage 4 *M. tuberculosis* strains from Guatemala

Strain	GenBank accession no.	Sequencing depth (×)	Genome size (bp)
GG-111-10	CP025593	539.6	4,411,563
GG-5-10	CP025594	496.0	4,411,442
GG-20-11	CP025595	403.2	4,411,504
GG-27-11	CP025596	565.5	4,411,443
GG-36-11	CP025597	557.2	4,411,469
GG-37-11	CP025598	530.7	4,411,526
GG-45-11	CP025599	464.7	4,411,469
GG-77-11	CP025600	627.0	4,411,508
GG-90-10	CP025601	522.9	4,411,602
GG-109-10	CP025602	550.3	4,411,463
GG-121-10	CP025603	526.2	4,411,510
GG-129-11	CP025604	525.2	4,411,413
GG-134-11	CP025605	551.0	4,411,399
GG-137-10	CP025606	475.6	4,411,446
GG-186-10	CP025607	327.6	4,411,478
GG-229-10	CP025608	557.2	4,411,519
